# Differences in bone density on chest CT according to smoking status in males without chronic obstructive lung disease

**DOI:** 10.1038/s41598-019-46830-4

**Published:** 2019-09-02

**Authors:** Cherry Kim, Soriul Kim, Ki Yeol Lee, Nan Hee Kim, Eun-Young Kang, Yu-Whan Oh, Chol Shin

**Affiliations:** 1 Department of Radiology, Ansan Hospital, Korea University College of Medicine, 123, Jeokgeum-ro, Danwon-gu, Ansan-si, Gyeonggi 15355 South Korea; 2 Institute for Human Genomic Study, College of Medicine, Korea University, 123, Jeokgeum-ro, Danwon-gu, Ansan-si, Gyeonggi 15355 South Korea; 3 Department of Endocrinology and Metabolism, Ansan Hospital, Korea University College of Medicine, 123, Jeokgeum-ro, Danwon-gu, Ansan-si, Gyeonggi 15355 South Korea; 4 Department of Radiology, Korea University Guro Hospital, Korea University College of Medicine, 148 Gurodong-ro, Guro-gu, Seoul, 08308 South Korea; 5 Department of Radiology, Anam Hospital, Korea University College of Medicine, 73 Inchon-ro, Seongbuk-gu, Seoul, 02841 South Korea; 6 Division of Pulmonary Sleep and Critical Care Medicine, Department of Internal Medicine, Ansan Hospital, Korea University College of Medicine, 123, Jeokgeum-ro, Danwon-gu, Ansan-si, Gyeonggi 15355 South Korea

**Keywords:** Computed tomography, Bone imaging

## Abstract

The goals of this study were to determine whether bone density measured using CT (CTBD) can show significant differences in bone loss according to smoking status and pack-years, and to examine the correlation between CTBD and bone mineral density when measured by dual-energy X-ray absorptiometry (DEXA-BMD) in males without chronic obstructive pulmonary disease (COPD). In this cross-sectional study, 1,011 males without airflow obstruction ≥50 years old were included. CTBD and DEXA-BMD were compared among groups with different smoking statuses. The correlation between CTBD and DEXA-BMD and the association of CTBD with pack-years were also investigated. CTBD of all vertebral bodies (VBs) and DEXA-BMD of all VBs without L1 showed significant differences among never, former, and current smokers. CTBD was significantly lowest in ≥30-pack-year smokers and was significantly lower in ≥30-pack-year smokers than in <15-pack-year smokers (all P < 0.05). There were significant correlations between DEXA-BMD and CTBD at all VB levels (correlation coefficient [r], 0.448~0.640; all P < 0.01). A lower CTBD had a significant association with a 15 ≤ x < 30-pack-year smoking history and ≥30-pack-year smoking history, while there was no association with never-smokers. In conclusion, CTBD demonstrated significant differences in bone quality according to smoking status and pack-years in males without COPD.

## Introduction

Smoking is a well-established risk factor that induces bone loss as a secondary cause of osteoporosis and increases the risk of fractures^1–3^. Therefore, it is very important that bone loss and osteoporosis are detected early in smokers. Meanwhile, most previous studies about the relationship between smoking and bone mineral loss have been performed using dual-energy X-ray absorptiometry (DEXA) only. However, several recent studies have shown that bone density measured on CT (CTBD) is also valuable for early detection of osteoporosis; it showed good correlation with bone mineral density measured by DEXA (DEXA-BMD) and good diagnostic performance for identifying bone fragility^4–7^.

There have been several studies investigating the relationship between smoking and bone mineral loss using CTBD. Male smokers, with or without chronic obstructive pulmonary disease (COPD), had a significant risk of low CTBD and vertebral fractures^8^. CTBD was significantly lower in smokers with emphysema and small airway disease on CT^9^. Lower values of CTBD were also found in current smokers compared to former smokers^10^. These studies were performed in COPD patients or in a general population that included COPD patients. However, COPD is a systemic condition affecting the development of osteoporosis, and the relationship between low CTBD and COPD has been reported in several studies^8,10^. Therefore, it is important to investigate the relationship between smoking and CTBD in a normal population without COPD, and no study has been published on this topic yet.

Therefore, the purpose of this study was to determine whether CTBD can show significant differences in bone quality according to smoking status and pack-years, and to examine the correlation between CTBD and DEXA-BMD in males ≥50 years old without COPD.

## Results

### CTBD and DEXA-BMD according to smoking status

Clinical characteristics, pulmonary function test (PFT), DEXA-BMD, CTBD, and emphysema volume on CT of the entire study cohort, participants <65 years old, and participants ≥65 years old are shown according to smoking status in Table 1. Among the three groups (the entire study cohort, participants <65 years old, and participants ≥65 years old), there were no significant differences in age, weight, body mass index (BMI), the number of regular exercisers, osteopenia, osteoporosis, and emphysema index (EI) between never-smokers and ever-smokers. However, never-smokers were significantly taller than ever-smokers in both the entire study cohort (P = 0.03) and in the group of participants <65 years old (P = 0.04). Furthermore, in all three groups, there were significantly more current drinkers and higher levels of alcohol consumption in ever-smokers than in never-smokers (all P < 0.001). FEV1/FVC (the ratio of forced expiratory volume in one second to forced vital capacity) was significantly lower in ever-smokers than in never-smokers in the three study groups, even after adjustment for age, height, and daily alcohol consumption (all P < 0.01).

**Table 1:** The clinical characteristics, pulmonary function test, bone mineral density measured by dual-energy X-ray absorptiometry (DEXA-BMD), and CT bone density (CTBD) of the entire study cohort, participants < 65 years old, and participants ≥ 65 years old, according to smoking status (never- and ever-smokers).

	Entire study cohort				<65 years old				≥65 years old			
	Never-smokers (n = 254)	Ever-smokers (n = 757)	P-value	P-value^a^	Never-smokers (n = 170)	Ever-smokers (n = 512)	P-value	P-value^a^	Never-smokers (n = 84)	Ever-smokers (n = 245)	P-value	P-value^a^
Clinical characteristics												
Age (years)	62.5 ± 7.2	62.1 ± 6.8	0.40	N/A	58.1 ± 3.0	58.0 ± 3.1	0.77	N/A	71.3 ± 4.5	70.5 ± 4.3	0.12	N/A
Height (cm)	166.6 ± 6.0	167.5 ± 5.5	0.03	N/A	167.3 ± 5.9	168.3 ± 5.4	0.04	N/A	165.4 ± 6.0	165.9 ± 5.2	0.44	N/A
Weight (kg)	67.9 ± 9.4	69.0 ± 9.1	0.09	N/A	69.4 ± 9.3	70.4 ± 9.1	0.23	N/A	64.8 ± 8.9	66.1 ± 8.4	0.22	N/A
BMI (kg/m^2^)	24.4 ± 2.9	24.6 ± 2.7	0.48	N/A	24.8 ± 2.9	24.8 ± 2.8	0.90	N/A	23.6 ± 2.6	24.0 ± 2.6	0.29	N/A
Regular exerciser, n	131 (51.2%)	370 (47.6%)	0.32	N/A	87 (51.2%)	252 (49.2%)	0.66	N/A	44 (52.4%)	109 (44.5%)	0.21	N/A
Current drinker, n	148 (57.8%)	550 (70.7%)	<0.001	N/A	105 (61.8%)	390 (76.2%)	<0.001	N/A	43 (51.2%)	145 (59.2%)	<0.001	N/A
Alcohol consumption (g/day)	7.9 ± 14.9	16.3 ± 24.0	<0.001	N/A	9.5 ± 15.9	23.4 ± 17.5	<0.001	N/A	4.6 ± 12.0	10.4 ± 20.0	<0.001	N/A
Pack-years	0	24.2 ± 18.3	<0.001	N/A	0	19.2 ± 25.2	<0.001	N/A	0	25.8 ± 20.0	<0.001	N/A
Osteoporosis and osteopenia, n	91 (35.8%)	306 (39.0%)	0.37	N/A	60 (35.3%)	197 (38.5%)	0.46	N/A	31 (36.9%)	98 (40.0%)	0.62	N/A
Osteopenia, n	70 (27.3%)	254 (32.7%)	0.25	N/A	49 (28.8%)	165 (32.2%)	0.71	N/A	21 (25.0%)	80 (32.7%)	0.24	N/A
Osteoporosis, n	21 (8.2%)	52 (6.7%)			11 (6.47%)	32 (6.25%)			10 (11.9%)	18 (7.4%)		
PFT												
FEV1 (L)	3.0 ± 0.5	3.0 ± 0.5	0.87	0.08	3.2 ± 0.5	3.1 ± 0.5	0.53	0.03	2.7 ± 0.4	2.7 ± 0.4	0.47	0.92
FVC (L)	3.9 ± 0.6	4.0 ± 0.6	0.03	0.32	4.0 ± 0.5	4.1 ± 0.6	0.25	0.83	3.6 ± 0.6	3.7 ± 0.5	0.02	0.05
FEV1/FVC ratio (%)	78.0 ± 4.9	76.1 ± 6.0	<0.001	<0.001	79.1 ± 4.0	77.4 ± 5.3	<0.001	<0.001	75.8 ± 5.8	73.1 ± 6.4	0.002	0.003
CTBD (HU)												
T4	202.7 ± 49.8	193.2 ± 45.8	0.005	0.002	208.9 ± 46.5	198.6 ± 44.0	0.009	0.006	190.0 ± 54.1	181.8 ± 47.3	0.19	0.15
T7	190.5 ± 45.9	184.2 ± 43.9	0.05	0.03	197.4 ± 41.4	191.2 ± 41.9	0.10	0.09	176.6 ± 51.5	169.6 ± 44.5	0.23	0.16
T10	192.4 ± 48.4	185.7 ± 44.5	0.04	0.03	202.2 ± 42.7	193.9 ± 42.7	0.03	0.03	172.7 ± 53.3	168.5 ± 43.3	0.52	0.46
L1	157.9 ± 42.6	148.9 ± 40.4	0.003	0.001	165.4 ± 38.1	157.5 ± 37.8	0.02	0.03	142.9 ± 47.1	131.0 ± 39.7	0.02	0.02
T4, T7, T10 (mean)	195.2 ± 45.9	187.7 ± 42.1	0.02	0.008	202.8 ± 41.2	194.6 ± 40.4	0.02	0.02	179.8 ± 5.0	173.3 ± 42.0	0.30	0.20
DEXA-BMD (g/cm^2^)												
L1	1.08 ± 0.20	1.06 ± 0.17	0.29	0.12	1.07 ± 0.17	1.06 ± 0.16	0.44	0.17	1.08 ± 0.24	1.06 ± 0.18	0.46	0.47
L2	1.17 ± 0.21	1.15 ± 0.19	0.19	0.05	1.17 ± 0.18	1.16 ± 0.18	0.58	0.19	1.19 ± 0.25	1.15 ± 0.20	0.18	0.17
L3	1.23 ± 0.21	1.22 ± 0.19	0.32	0.12	1.23 ± 0.19	1.22 ± 0.18	0.49	0.16	1.25 ± 0.26	1.15 ± 0.20	0.48	0.51
L4	1.28 ± 0.23	1.27 ± 0.21	0.43	0.17	1.27 ± 0.21	1.26 ± 0.21	0.62	0.20	1.30 ± 0.27	1.28 ± 0.23	0.51	0.58
L1-L4 (mean)	1.12 ± 0.20	1.18 ± 0.19	0.15	0.03	1.20 ± 0.18	1.18 ± 0.18	0.27	0.03	1.23 ± 0.25	1.20 ± 0.21	0.37	0.47
L2-L4 (mean)	1.24 ± 0.21	1.22 ± 0.19	0.14	0.05	1.23 ± 0.19	1.21 ± 0.18	0.28	0.07	1.27 ± 0.26	1.23 ± 0.21	0.34	0.40
Emphysema volume in CT												
Emphysema index (%)	3.74 ± 2.62	3.87 ± 2.78	0.51	0.29	3.74 ± 2.50	3.79 ± 2.74	0.82	0.71	3.74 ± 2.86	4.03 ± 2.86	0.42	0.19

^a^P-values from ANOVA after adjusting for age, height, and daily alcohol consumption.

Note- N/A, not applicable; BMI, body mass index; PFT, pulmonary function test; DEXA-BMD, bone mineral density measured by dual-energy X-ray absorptiometry; CTBD, CT bone density; FEV1, forced expiratory volume in one second; FVC, forced vital capacity; FEV1/FVC, ratio of FEV1 to FVC.

CTBD of all vertebral bodies (VB) was significantly lower in ever-smokers than in never-smokers in the entire study cohort, after adjustment (all P < 0.05). Among participants <65 years old, CTBD of the T10 and L1, and mean CTBD of T4, T7, and T10 were significantly lower in ever-smokers than in never-smokers, after adjustment (all P < 0.05). Among participants ≥65 years old, only CTBD of L1 was significantly lower in ever-smokers than in never-smokers, after adjustment (P = 0.02). In the multivariate linear regression analyses, among participants ≥65 years old, there was no significant association between smoking status and mean CTBD of T4, T7, and T10; however, age itself showed a significant association with mean CTBD of T4, T7, and T10 (β [standard error, SE], −2.05 [0.60]; P < 0.001). Both smoking status and age were associated with CTBD of L1 (β [SE], −13.82 [5.75] and −1.92 [0.57]; all P < 0.05) (Supplementary Table S1).

There were no significant differences in DEXA-BMD values in any VBs between never-smokers and ever-smokers in the entire study cohort, as well as among participants <65 years old and ≥65 years old, before or after adjustment.

Comparisons of the clinical characteristics, PFT, DEXA-BMD, and CTBD between never, former, and current smokers are shown in Table 2. The trends that CTBD and DEXA-BMD of current smokers were lower than those of former and never smokers, and that CTBD and DEXA-BMD of former smokers were lower than those of never smokers were shown in all VBs, although current smokers were significantly younger than former and never smokers. After adjustment of covariates, CTBD of all VBs and all DEXA-BMD values without DEXA-BMD of L1 showed significant differences between never, former, and current smokers.

**Table 2:** Clinical characteristics, pulmonary function test, bone mineral density measured by dual-energy X-ray absorptiometry (DEXA-BMD), and CT bone density (CTBD) of the entire study cohort according to smoking status (never, former, and current smokers).

	Never smokers (n = 254)	Former smokers (n = 568)	Current smokers (n = 189)	P-value	Adjusted P-value^a^
Clinical characteristics					
Age (years)	62.5 ± 7.2	62.8 ± 7.0	59.8 ± 5.7	<0.001	N/A
Height (cm)	166.7 ± 6.0	167.4 ± 5.5	167.9 ± 5.2	0.06	N/A
Weight (kg)	67.9 ± 9.4	69.1 ± 8.8	68.7 ± 9.8	0.21	N/A
BMI (kg/m^2^)	24.4 ± 2.9	24.7 ± 2.6	24.3 ± 3.0	0.28	N/A
Regular exerciser, n	131 (51.6%)	282 (49.7%)	79 (41.8%)	0.10	N/A
Current drinker, n	148 (58.3%)	388 (68.3%)	147 (77.8%)	<0.001	N/A
Alcohol consumption (g/day)	7.9 ± 14.9	14.5 ± 21.7	21.9 ± 29.2	<0.001	N/A
Osteopenia, n	70 (27.6)	179 (31.5)	66 (34.9)	0.006	N/A
Osteoporosis, n	21 (8.3)	28 (4.9)	22 (11.6)		
PFT					
FEV1 (L)	3.0 ± 0.5	3.0 ± 0.5	3.0 ± 0.5	0.87	0.001
FVC (L)	3.9 ± 0.6	4.0 ± 0.6	4.0 ± 0.6	0.09	0.41
FEV1/FVC ratio (%)	78.0 ± 4.9	76.2 ± 5.6	75.6 ± 7.0	<0.001	<0.001
CTBD (HU)					
T4	202.7 ± 49.8	193.5 ± 47.1	192.2 ± 41.6	0.02	0.003
T7	190.5 ± 45.9	183.8 ± 44.8	183.2 ± 41.5	0.14	0.04
T10	192.4 ± 48.4	185.1 ± 45.7	184.2 ± 40.6	0.11	0.03
L1	157.9 ± 42.6	149.1 ± 40.9	148.4 ± 39.0	0.01	0.001
T4, T7, T10 (mean)	195.20 ± 45.9	187.5 ± 43.3	188.2 ± 38.2	0.05	0.008
DEXA-BMD (g/cm^2^)					
L1	1.08 ± 0.20	1.07 ± 0.17	1.03 ± 0.17	0.01	0.05
L2	1.17 ± 0.21	1.17 ± 0.19	1.12 ± 0.17	0.005	0.006
L3	1.23 ± 0.21	1.23 ± 0.19	1.18 ± 0.19	0.009	0.04
L4	1.28 ± 0.23	1.28 ± 0.21	1.22 ± 0.20	0.001	0.009
L1-L4 (mean)	1.20 ± 0.21	1.20 ± 0.19	1.15 ± 0.18	0.006	0.02
L2-L4 (mean)	1.24 ± 0.21	1.23 ± 0.20	1.18 ± 0.18	0.005	0.01

^a^P-values from ANOVA after adjusting for age, height, and daily alcohol consumption.

Note- N/A, not applicable; BMI, body mass index; PFT, pulmonary function test; DEXA-BMD, bone mineral density measured by dual-energy X-ray absorptiometry; CTBD, CT bone density; FEV1, forced expiratory volume in one second; FVC, forced vital capacity; FEV1/FVC, ratio of FEV1 to FVC.

### CTBD and DEXA-BMD according to pack-years

**Figure 1:**
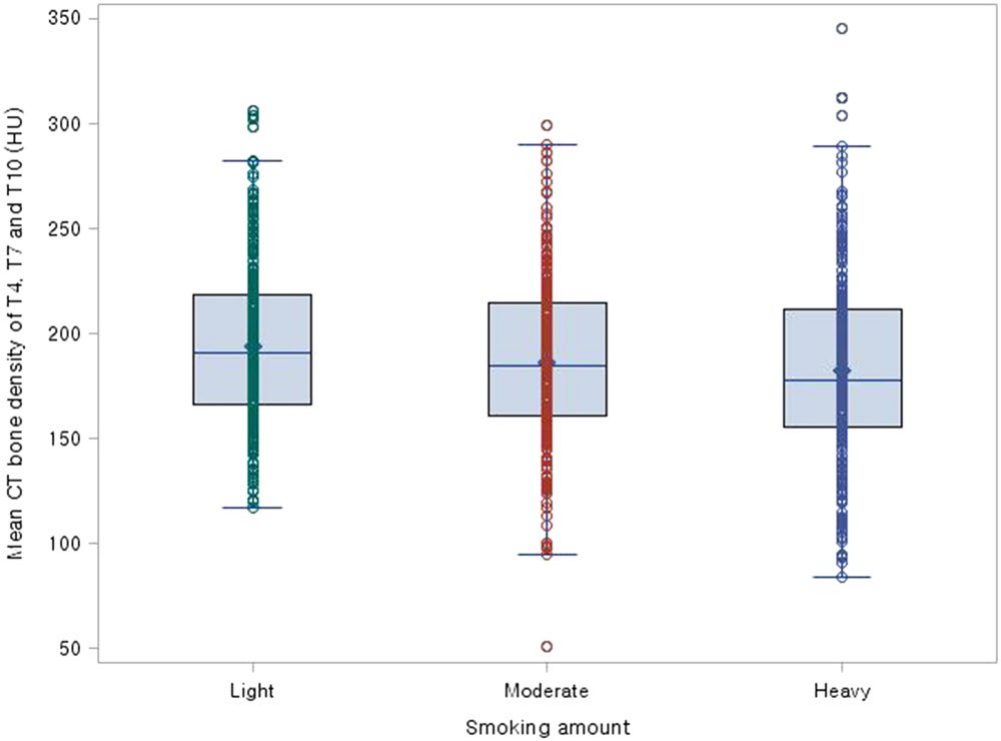
Mean CT bone density values of T4, T7, and T10 of the ever-smoker group with different pack-years (G1 [light], <15 pack-years; G2 [moderate], 15 ≤ x < 30 pack-years; and G3 [heavy], ≥30 pack-years).

Clinical characteristics, PFT, DEXA-BMD, and CTBD between the groups with different pack-years (light smokers, pack-years <15; moderate smokers, 15 ≤ x < 30 pack-years; and heavy smokers, pack-years ≥30) in ever-smokers are shown in Table 3 and Fig. 1. There were no significant differences in age, height, weight, BMI, number of regular exercisers or current drinkers, osteopenia, or osteoporosis between the groups. However, there were significantly higher levels of alcohol consumption in heavy smokers than in moderate and light smokers (all P < 0.05). Among the pack-years groups, FEV1 (forced expiratory volume in one second) and FVC (forced vital capacity) were significantly lowest in heavy smokers, after adjustment for age, height, and daily alcohol consumption (all P < 0.05).

**Table 3:** The clinical characteristics, pulmonary function test, bone mineral density measured by dual-energy X-ray absorptiometry (DEXA-BMD), and CT bone density (CTBD) of the ever-smoker groups with different pack-years (light smokers, <15 pack-years; moderate smokers, 15 ≤ x < 30 pack-years; and heavy smokers, ≥30 pack-years).

	0< pack-years <15 (n = 259)	15≤ pack-years <30 (n = 238)	≥30 pack-years (n = 260)	P-value	Adjusted P-value^a^
Clinical characteristics					
Age (years)	61.7 ± 6.7	61.9 ± 6.7	62.5 ± 7.0	0.37	N/A
Height (cm)	167.1 ± 5.3	167.3 ± 6.0	168.1 ± 5.2	0.11	N/A
Weight (kg)	68.6 ± 8.8	69.0 ± 9.1	69.5 ± 9.3	0.48	N/A
BMI (kg/m^2^)	24.5 ± 2.6	24.6 ± 2.6	24.6 ± 2.9	0.93	N/A
Regular exerciser, n	133 (51.4%)	119 (50.0%)	109 (41.9%)	0.07	N/A
Current drinker, n	187 (72.2%)	170 (71.4%)	178 (68.5%)	0.78	N/A
Alcohol consumption (g/day)	13.9 ± 20.0	14.4 ± 20.8	20.4 ± 29.4	0.003^†‡^	N/A
Osteopenia, n	84 (32.4%)	71 (29.8%)	90 (34.6%)	0.22	N/A
Osteoporosis, n	12 (4.6%)	15 (6.3%)	23 (8.9%)		
PFT					
FEV1 (L)	3.1 ± 0.4	3.0 ± 0.5	2.9 ± 0.5	0.01^‡^	<0.001
FVC (L)	4.0 ± 0.5	3.9 ± 0.6	3.9 ± 0.6	0.07	0.001
FEV1/FVC ratio (%)	76.3 ± 5.5	76.5 ± 5.7	75.4 ± 6.7	0.09	0.21
CTBD (HU)					
T4	197.3 ± 43.2	193.1 ± 45.1	189.1 ± 48.5	0.13	0.21
T7	190.0 ± 40.8	182.3 ± 42.9	180.1 ± 47.3	0.03^‡^	0.07
T10	193.7 ± 41.3	184.2 ± 43.2	178.9 ± 47.4	<0.001^‡^	0.003
L1	156.2 ± 38.0	148.1 ± 38.6	142.4 ± 43.1	<0.001^‡^	0.002
T4, T7, T10 (mean)	193.7 ± 38.8	186.6 ± 41.2	182.7 ± 45.2	0.01^‡^	0.03
DEXA-BMD (g/cm^2^)					
L1	1.07 ± 0.16	1.07 ± 0.17	1.05 ± 0.17	0.18	0.05
L2	1.16 ± 0.18	1.16 ± 0.19	1.14 ± 0.19	0.17	0.04
L3	1.23 ± 0.19	1.22 ± 0.19	1.21 ± 0.20	0.55	0.21
L4	1.27 ± 0.20	1.27 ± 0.22	1.26 ± 0.22	0.72	0.33
L1-L4 (mean)	1.19 ± 0.18	1.19 ± 0.19	1.17 ± 0.19	0.50	0.59
L2-L4 (mean)	1.22 ± 0.19	1.22 ± 0.19	1.20 ± 0.20	0.49	0.25

^†^P < 0.05 when comparing G2 to G3 by Bonferroni correction.

^‡^P < 0.05 when comparing G1 to G3 by Bonferroni correction.

^a^Adjusted P-values by ANOVA after adjusting for age, height, and daily alcohol consumption.

Note- N/A, not applicable; BMI, body mass index; PFT, pulmonary function test; DEXA-BMD, bone mineral density measured by dual-energy X-ray absorptiometry; CTBD, CT bone density; FEV1, forced expiratory volume in one second; FVC, forced vital capacity; FEV1/FVC, ratio of FEV1 to FVC.

Among the pack-years groups, the CTBD of T7, T10, and L1, and mean CTBD of T4, T7, and T10 were significantly lowest in the heavy smokers group, and were also significantly lower in heavy smokers than in light smokers (all P < 0.05). After adjustment, CTBD of the T10 and L1, and mean CTBD of T4, T7, and T10 were significantly lowest in heavy-smokers (all P < 0.05). However, only the DEXA-BMD of L2 showed a significant difference between the groups after adjustment (P = 0.04).

The associations of CTBD of all VBs with pack-years on multiple linear regression analysis are shown in Table 4. In all VBs, a lower CTBD value was significantly associated with moderate smokers (β [SE] values of CTBD of T4, T7, T10, and L1, and mean CTBD of T4, T7, and T10 were −10.6 [4.2], −9.0 [3.9], −9.0 [3.9], −10.5 [3.5], and −9.5 [3.8], respectively; all P < 0.05) and heavy smokers (β [SE] values of CTBD of T4, T7, T10, and L1, and mean CTBD of T4, T7, and T10 were −14.6 [4.1], −10.0 [3.9], −12.7 [3.9], −14.6 [3.5], and −12.3 [3.7], respectively; all P < 0.01) when compared with never smokers.

**Table 4:** The association of CT bone density (CTBD) of all vertebral bodies with pack-years (never-smokers; light smokers, <15 pack-years; moderate smokers, 15 ≤ x < 30 pack-years; and heavy smokers, ≥30 pack-years) on multiple linear regression analysis.

CTBD (HU)	Never-smokers (n = 254)	0< pack-years <15 (n = 259)		15≤ pack-years <30 (n = 238)		≥30 pack-years (n = 260)		Trend P-value
		${\rm{\beta }}$ (SE)	P-value	${\rm{\beta }}$ (SE)	P-value	${\rm{\beta }}$ (SE)	P-value	
T4	Reference	−6.8 (4.1)	0.1	−10.6 (4.2)	0.01	−14.0 (4.1)	<0.001	<0.001
T7	Reference	−1.8 (3.8)	0.63	−9.0 (3.9)	0.02	−10.0 (3.9)	0.01	0.002
T10	Reference	−0.1 (3.8)	0.97	−9.0 (3.9)	0.02	−12.7 (3.9)	0.001	<0.001
L1	Reference	−3.0 (3.4)	0.38	−10.5 (3.5)	0.003	−14.6 (3.5)	<0.001	<0.001
T4, T7, T10 (mean)	Reference	−2.9 (3.7)	0.43	−9.5 (3.8)	0.01	−12.3 (3.7)	0.001	<0.001

All P-values were based on linear regression analysis on CTBD of all VBs and adjusted for age, height, and daily alcohol consumption.

Note- CTBD, CT bone density; SE, standard error.

### Correlations between CTBD and DEXA-BMD

Table 5 shows the correlations between DEXA-BMD at each VB, CTBD at each VB, and pack-years in the entire study cohort, in never-smokers, and in ever-smokers. There were significant correlations between DEXA-BMD and CTBD at all VB levels among the whole study cohort (correlation coefficient [r], 0.448~0.640), never-smokers (r, 0.474~0.643), and ever-smokers (r, 0.427~0.651) (all P < 0.01), after adjustment for age, height, and daily alcohol consumption. Pack-years also had a significant negative correlation with DEXA-BMD and CTBD at all VBs in the whole study cohort (r, −0.030 ~ −0.022; P < 0.01) and in ever-smokers (r, −0.023 ~ −0.145; P < 0.01), after adjustment.

**Table 5:** The correlations between the value of bone mineral density measured by dual-energy X-ray absorptiometry at each vertebral body, CT bone density at each vertebral body, and pack-years in the whole study cohort, never-smokers, and ever-smokers.

	DEXA- BMD (L1)	DEXA- BMD (L2)	DEXA- BMD (L3)	DEXA- BMD (L4)	DEXA- BMD (L1-L4, mean)	DEXA- BMD (L2-L4, mean)	CTBD (T4, T7, T10 [mean])	CTBD (T4)	CTBD (T7)	CTBD (T10)	CTBD (L1)	Pack-years
	*Total* (*N* = *1011*)											
DEXA-BMD (L1)	1.000	0.916**	0.878**	0.817**	0.942**	0.905**	0.588**	0.640**	0.520**	0.503**	0.563**	−0.067**
DEXA-BMD (L2)		1.000	0.933**	0.862**	0.967**	0.961**	0.567**	0.617**	0.504**	0.484**	0.552**	−0.067**
DEXA-BMD (L3)			1.000	0.919**	0.977**	0.982**	0.572**	0.621**	0.507**	0.490**	0.545**	−0.043*
DEXA-BMD (L4)				1.000	0.961**	0.972**	0.531**	0.582**	0.472**	0.448**	0.489**	−0.030*
DEXA-BMD												
(L1-L4, mean)					1.000	0.996**	0.606**	0.647**	0.557**	0.518**	0.578**	−0.069**
DEXA-BMD												
(L2-L4, mean)						1.000	0.598**	0.637**	0.549**	0.511**	0.568**	−0.067**
CTBD												
(T4, T7, T10 [mean])							1.000	0.935**	0.954**	0.945**	0.856**	−0.115**
CTBD (T4)								1.000	0.836**	0.809**	0.789**	−0.103**
CTBD (T7)									1.000	0.871**	0.806**	−0.094*
CTBD (T10)										1.000	0.833**	−0.129**
CTBD (L1)											1.000	−0.155**
	*Never smokers* (*N* = *254*)											
DEXA-BMD (L1)	1.000	0.927**	0.895**	0.827**	0.943**	0.901**	0.587**	0.643**	0.519**	0.514**	0.592**	N/A
DEXA-BMD (L2)		1.000	0.949**	0.884**	0.974**	0.968**	0.543**	0.598**	0.481**	0.474**	0.548**	N/A
DEXA-BMD (L3)			1.000	0.921**	0.978**	0.982**	0.570**	0.616**	0.503**	0.511**	0.551**	N/A
DEXA-BMD (L4)				1.000	0.960**	0.972**	0.556**	0.595**	0.496**	0.500**	0.497**	N/A
DEXA-BMD (L1-L4, mean)					1.000	0.995**	0.586**	0.615**	0.542**	0.526**	0.555**	N/A
DEXA-BMD (L2-L4, mean)						1.000	0.573**	0.599**	0.531**	0.516**	0.536**	N/A
CTBD (T4, T7, T10 [mean])							1.000	0.946**	0.961**	0.958**	0.877**	N/A
CTBD (T4)								1.000	0.858**	0.846**	0.840**	N/A
CTBD (T7)									1.000	0.900**	0.829**	N/A
CTBD (T10)										1.000	0.845**	N/A
CTBD (L1)											1.000	N/A
	*Ever smokers* (*N* = *757*)											
DEXA-BMD (L1)	1.000	0.912**	0.870**	0.813**	0.942**	0.907**	0.588**	0.638**	0.519**	0.497**	0.552**	−0.068**
DEXA-BMD (L2)		1.000	0.927**	0.853**	0.964**	0.958**	0.575**	0.623**	0.512**	0.485**	0.553**	−0.061*
DEXA-BMD (L3)			1.000	0.918**	0.977**	0.982**	0.572**	0.623**	0.508**	0.480**	0.542**	−0.036
DEXA-BMD (L4)				1.000	0.962**	0.972**	0.521**	0.578**	0.463**	0.427**	0.486**	−0.023
DEXA-BMD (L1-L4, mean)					1.000	0.996**	0.613**	0.659**	0.561**	0.512**	0.585**	−0.057
DEXA-BMD (L2-L4, mean)						1.000	0.606**	0.651**	0.554**	0.506**	0.579**	−0.055*
CTBD (T4, T7, T10 [mean])							1.000	0.930**	0.951**	0.940**	0.847**	−0.104*
CTBD (T4)								1.000	0.827**	0.794**	0.767**	−0.078
CTBD (T7)									1.000	0.860**	0.796**	−0.085
CTBD (T10)										1.000	0.827**	−0.133**
CTBD (L1)											1.000	−0.145**

**P < 0.01, *P < 0.05.

All data were adjusted for age, height, and daily alcohol consumption.

Note- N/A, not applicable; DEXA-BMD, bone mineral density measured by dual-energy X-ray absorptiometry; CTBD, CT bone density.

## Discussion

This study provided evidence that smoking is associated with decreased bone density measured by chest CT in male subjects ≥50 years old without COPD. Current smokers showed significantly lower bone densities than former and never smokers. Lower bone density on CT was significantly associated with more pack-years. CTBD also showed a significant correlation with DEXA-BMD, and CTBD and DEXA-BMD were significantly correlated with pack-years.

In many previous studies, CTBD has been shown to be an effective method of demonstrating bone mineral loss and has shown good correlation with DEXA-BMD^1,5,7,9,11–16^. Recently, it was also reported that CTBD reflects bone mineral loss more sensitively than DEXA, although CTBD measures were calibrated using specific formulas^12^. In our study, we showed that CTBD of the T4, T7, T10, and L1, and mean CTBD of T4, T7, and T10 were all significantly lower in ever-smokers than in never-smokers among males ≥50 years old without COPD. In ever-smokers, the significantly lowest CTBD of T10 and L1, and mean CTBD of T4, T7, and T10 were shown in heavy smokers after adjustment for covariates. However, there were no significant differences in the DEXA-BMD values of any VBs between never-smokers and ever-smokers. Also, all CTBD values and some of the DEXA-BMD values of current smokers were significantly lower than those of former and never smokers; however, DEXA-BMD of L1 did not show significant differences between the groups. In addition, the lower CTBD of the thoracic and lumbar VBs in ever-smokers was associated with higher pack-years, although only the DEXA-BMD of L2 was significantly lowest in heavy smokers among ever-smokers. This may suggest that CTBD is a more sensitive method than DEXA-BMD.

There have only been a few studies dealing with the association between smoking and bone mineral loss in normal subjects without COPD. Since COPD is one of the causes of osteoporosis^17–21^, excluding COPD patients is considered a more accurate analysis of the relationship between smoking and bone mineral loss without bias. In a cross-sectional study of Japanese men aged ≥65 years, DEXA-BMD of the lumbar spine and total hip decreased with increasing numbers of pack-years or numbers of smoking years^22^. However, in this study, bone mineral density was measured by DEXA only, and this research was conducted using the lumbar spine, not the thoracic spine. Another study demonstrated that current smokers showed more rapid bone mineral loss using CTBD over a 3-year period compared with former smokers, which correlates with our results^23^.

In our study, DEXA-BMD values were not significantly different between never-smokers and ever-smokers. A previous study also showed that bone mineral density of the lumbar spine, femur neck, and total hip measured by DEXA between ever-smokers and never-smokers did not differ significantly, although pack-years of smoking showed a negative correlation with total hip DEXA-BMD in the ever-smoker group^24^. In another previous DEXA study, no significant decreases of bone mineral density were found in most aging groups^12^. These results are due to measurement of bone density by distinguishing between trabecular and cortical bone in CT. Trabeculae should be differentiated from the cortical bone because they are more sensitive to bone metabolism than cortical bone, and are therefore a better site for determining bone mineral loss^6,25–27^. In addition, DEXA-BMD of the lumbar spine in the elderly is well known to suffer measurement difficulties due to deformities, fractures, or aortic calcifications, which affect the values as artifacts^2,28^. The advantage of CTBD is that it avoids these artifacts and thus can provide a more accurate value for bone mineral loss.

Since the national lung cancer screening trial (NLST) demonstrated that annual low-dose chest CT screening for individuals at high risk of lung cancer reduced relative mortality by 20%^29^, an increasing need for chest CT screening and surveillance of pulmonary nodules in smokers has emerged. Based on our results, if additional information on the bone density of the thoracic and lumbar spine in CT screening for smokers could be given, it would help detect not only lung nodules but also bone mineral loss at an earlier stage. Therefore, chest CT screening for smokers will increase opportunities for identifying and treating osteoporosis in this at-risk population.

Since bone mineral loss becomes severe with aging, in order to distinguish the influences of aging on bone mineral loss, we conducted an additional analysis by dividing the study population into two groups: participants <65 years old and participants ≥65 years old. In participants <65 years old, ever-smokers showed significantly lower CTBD of the T10 and L1 VBs and lower mean CTBD of T4, T7, and T10 than never-smokers. In participants ≥65 years old, only the CTBD of the L1 VB was significantly lower in ever-smokers than in never-smokers. These results were due to the effects of aging rather than smoking on bone mineral loss, as shown in multivariate linear regression analyses.

Our study has several limitations, being that it is a cross-sectional study. First, the sample size of never-smokers was relatively small compared with ever-smokers. Also, this cohort was recruited from a specific area in Korea, which is characterized mainly by industrial complexes. These subjects might not be a representative sample of the whole nation, and further studies with a larger sample population are required to clarify those uncertainties. Second, DEXA-BMD was measured only for the lumbar spine, while CTBD was mainly measured for the thoracic spine. However, DEXA usually does not measure the thoracic spine, and correlation analysis in our study revealed a significant correlation between CTBD and DEXA-BMD. Third, we did not investigate the prevalence of vertebral fractures, because these data were not in available in this cohort. In addition, we measured bone density at only one point, based on which it was difficult to predict the future potential of vertebral fracture. In future studies, the correlation between the prevalence of CTBD and vertebral fractures should be studied using longitudinal data. Fourth, data regarding pack-years or alcohol consumption were entirely self-reported, and this might cause under- or over-estimation of the smoking effect. However, trained staff conducted all interviews for quality control. Finally, because one radiologist measured all CTBD values, the variability of measured values between observers (i.e., interobserver-variability) could not be analyzed.

In conclusion, CTBD showed significant differences in bone quality according to smoking status and pack-years in males ≥50 years old without COPD. Significantly lower CTBD were shown in ever-smokers than in never-smokers, and lower CTBD was associated with more pack-years. Current smokers showed significantly lower CTBD than former and never smokers. Also, CTBD was significantly correlated with DEXA-BMD.

## Methods

### Study participants and data collection

This retrospective cross-sectional study was approved by the institutional review board of Korea University Ansan Hospital (approval number: 2006AS0045) and the requirement for informed consent was waived. This cross-sectional study used data from the Ansan cohort included in the Korean Genome and Epidemiology Study (KoGES). The profile of the cohort has been described in detail elsewhere^6^. Between 2001 and 2002, a total of 5,012 participants were examined as a baseline, and follow-up examinations have been performed biennially. The eighth set of follow-up examinations was performed in 3,083 individuals between April 2015 and December 2016, and of these, 1,334 males ≥50 years old underwent both chest CT and DEXA. The exclusion criteria were: a) underlying lung disease such as COPD (FEV1/FVC <0.70) or pulmonary tuberculosis, b) taking anti-osteoporotic medications affecting bone metabolism, and c) history of malignancy. Finally, 1,011 subjects were selected for the analysis.

All participants were interviewed by trained health nurses or medical doctors to collect data on sex, age, smoking status (never-/ever-smokers or never/former/current-smokers), pack-years (never-smokers; light smokers, <15 pack-years; moderate smokers, 15 ≤ x < 30 pack-years; and heavy smokers, ≥30 pack-years), current drinking status, amount of daily alcohol consumption, regular exercise, and medical conditions. Trained staff measured the height and weight of each participant using standard protocols, and BMI was calculated.

### CT acquisition and image reconstruction

All CT images were acquired with one 64-channel multi-detector CT scanner (Brilliance 64; Cleveland, USA, Philips)^30^. The following CT scanning parameters were employed: detector configuration, 64 × 0.625 mm; rotation time, 0.5 sec; tube voltage, 120 kVp; tube current, 75 mAs without current modulation; and section thickness, 0.625 mm. Contrast enhancement was not performed. Each subject was examined in the supine position with a deep inspiratory breath hold.

### Measurement of CTBD

One radiologist (C.K.) measured the CT attenuation value (HU) of the T4, T7, T10, and L1 in each chest CT to obtain CTBD value. The measurements were performed according to previous studies^4,7,9,23^. A region of interest (ROI) was manually placed in the axial plane of each VB in the upper part of the VB between the endplate and the entrance of the vessels at the anterior mid portion. Volumetric analysis was not performed, as CTBD was shown to be sensitive to bone mineral loss even by measuring in 2D axial images without volumetric analysis in previous studies^4,7,9,23^. In addition, the previous study concluded that vertebral attenuation values can be manually quantified with good to excellent inter-examination and inter-observer reliability^4^. Inhomogeneous areas showing cortical bone, areas with large vessels, bone islands, fractures, and calcified herniated discs were avoided, and ROIs were as large as possible. If the VB was fractured, inhomogeneous, or not visualized, an ROI was placed in an adjacent vertebra. Height loss of more than 20% compared to an adjacent non-fractured vertebra was considered a fracture according to the previous report^23,27^. Mean density of the T4, T7, and T10 was also calculated.

### DEXA-BMD

DEXA-BMD was measured at the lumbar spine, including L1, L2, L3, and L4, and the mean values of L1 to L4 and L2 to L4 were also calculated using DEXA (Lunar Prodigy Advance, GE Healthcare, Madison, WI). The diagnoses of osteoporosis and osteopenia were based on the criteria of the World Health Organization (WHO) and each patient’s lowest T-score of the three DEXA measurements. Osteoporosis was defined as a T-score ≤ −2.5 standard deviations (SD), and osteopenia was defined as a T-score between −1.0 SD and −2.5 SD, as defined in the WHO criteria^26^.

### Pulmonary function test

A PFT was performed within one week prior to referral for CT scanning. Forced spirometry was performed by a skilled technician using a spirometer (Vmax-2130, Viasys Healthcare, Yorba Linda, CA, USA), according to the American Thoracic Society (ATS)/European Respiratory Society (ERS) criteria for standardization^27^. PFT parameters are expressed in liters and percentages, and the following spirometric values were investigated: FEV1, FVC, and FEV1/FVC after bronchodilation.

### Emphysema volume in CT

For quantitative analysis of emphysema volume, all CT images were analyzed with commercial software (IntelliSpace Portal 7.0 Philips Healthcare, Cleveland, OH, USA). This tool performs 4 steps: (1) lung segmentation, (2) lung density measurement, (3) airway extraction, and (4) airway measurement. Total lung volume and emphysema volume with a threshold of −950 HU were measured, then EI were automatically obtained.

### Statistical analysis

All continuous variables are expressed as mean ± SD and categorical variables are expressed as percentages. Clinical characteristics, PFT, DEXA-BMD, CTBD, and EI were compared between smoking status groups (never/ever-smokers and never/former/current smokers) in the entire study cohort, as well as in participant groups <65 years and ≥65 years. Pearson correlations were calculated between DEXA-BMD, CTBD, and pack-years in the whole study cohort, never-smokers, and ever-smokers. In ever-smokers, clinical characteristics, PFT, DEXA-BMD, and CTBD were also compared between groups having different pack-years using the one-way analysis of variance (ANOVA) or Chi-squared test. Bonferroni’s post hoc test for multiple comparisons was performed. Multivariate linear regression analyses were performed, and the regression coefficient (β) and SE were calculated to assess the association of aging with CTBD and the association of CTBD with pack-years. In all analyses, P-values were adjusted for age, height, and daily alcohol consumption. Those statistical analyses were performed using SAS version 9.4 (SAS Institute, Cary, NC, USA). All P-values < 0.05 were considered statistically significant.

## Supplementary information

Supplementary Table S1

## Data Availability

The data in this study are based on a strictly managed cohort from the Korea National Institute of Health (KNIH), South Korea, and cannot be disclosed without permission.
