# Supplementary material for: Differences in bone density on chest CT according to smoking status in males without chronic obstructive lung disease
Source: Sci Rep. 2019 Sep 2;9:10467. doi: 10.1038/s41598-019-46830-4 (PMC6718668; doi:10.1038/s41598-019-46830-4)
Supplement: Supplementary file 1 — Supplementary Table S1 [file 41598_2019_46830_MOESM1_ESM.docx]

**Differences in** **bone density on chest CT according to smoking status in males without chronic obstructive lung disease**

Cherry Kim^+^, MD, PhD^1^, Soriul Kim^+^, PhD^2^, Ki Yeol Lee*, MD, PhD^1^, Nan Hee Kim, MD, PhD^3^, Eun-Young Kang, MD, PhD^4^, Yu-Whan Oh, MD, PhD^5^, Chol Shin, MD, PhD^6^

^+^Cherry Kim and Soriul Kim contributed equally to this work.

^1^Department of Radiology, Ansan Hospital, Korea University College of Medicine, 123, Jeokgeum-ro, Danwon-gu, Ansan-si, Gyeonggi, 15355, South Korea

^2^Institute for Human Genomic Study, College of Medicine, Korea University, 123, Jeokgeum-ro, Danwon-gu, Ansan-si, Gyeonggi, 15355, South Korea

^3^Department of Endocrinology and Metabolism, Ansan Hospital, Korea University College of Medicine, 123, Jeokgeum-ro, Danwon-gu, Ansan-si, Gyeonggi, 15355, South Korea

^4^Department of Radiology, Korea University Guro Hospital, Korea University College of Medicine, 148 Gurodong-ro, Guro-gu, Seoul, 08308, South Korea

^5^Department of Radiology, Anam Hospital, Korea University College of Medicine, 73 Inchon-ro, Seongbuk-gu, Seoul, 02841, South Korea

^6^Division of Pulmonary Sleep and Critical Care Medicine, Department of Internal Medicine, Ansan Hospital, Korea University College of Medicine, 123, Jeokgeum-ro, Danwon-gu, Ansan-si, Gyeonggi, 15355, South Korea

^*^**Corresponding author:** Ki Yeol Lee, MD, PhD

Department of Radiology, Ansan Hospital, Korea University College of Medicine, 516, Gojan 1-dong, Danwon-gu, Ansan-si, Gyeonggi, South Korea.

E-mail: [kiylee@korea.ac.kr](mailto:kiylee@korea.ac.kr)

Supplementary Table S1. The associations of CT bone density (CTBD) of mean value of T4, T7, and T10, and L1 with independent variables using multivariate linear regression analyses in the entire study cohort, in participants <65 years old, and in participants ≥65 years old.

| **Independent**  **Variables** | **Entire study cohort** | | | | | |
| --- | --- | --- | --- | --- | --- | --- |
|  | **CTBD of T4, T7, T10 (mean)** | | | **CTBD of L1** | | |
|  | **Unadjusted** | **Model 1** | **Model 2** | **Unadjusted** | **Model 1** | **Model 2** |
|  | **β (SE)** | **β (SE)** | **β (SE)** | **β (SE)** | **β (SE)** | **β (SE)** |
| Smoking  status | -7.52 (3.12)  (P=0.02) | -8.26 (3.00)  (P=0.006) | -9.90 (3.33)  (P=0.003) | -8.94 (2.98)  (P <0.001) | -9.81 (2.81)  (P <0.001) | -10.74 (3.11)  (P <0.001) |
| Age |  | -1.72 (0.19)  (P <0.001) | -1.78 (0.21)  (P <0.001) |  | -2.01 (0.18)  (P <0.001) | -2.07 (0.19)  (P <0.001) |
| Height |  |  | -0.17 (0.26)  (P=0.51) |  |  | -0.23 (0.24)  (P=0.35) |
| Alcohol consumption |  |  | -0.02 (0.07)  (P=0.74) |  |  | -0.06 (0.06)  (P=0.29) |
| Emphysema index (%) |  |  | -0.73 (0.51)  (P=0.15) |  |  | -0.49 (0.48)  (P=0.31) |
| **Independent**  **Variables** | **<65 years old** | | | | | |
|  | **Unadjusted** | **Model 1** | **Model 2** | **Unadjusted** | **Model 1** | **Model 2** |
|  | **β (SE)** | **β (SE)** | **β (SE)** | **β (SE)** | **β (SE)** | **β (SE)** |
| Smoking  status | -8.26 (3.11)  (P<0.001) | -8.39 (3.57)  (P=0.02) | -10.65 (3.98)  (P=0.008) | -7.94 (3.38)  (P<0.001) | -7.94 (3.32)  (P=0.02) | -9.02 (3.68)  (P=0.01) |
| Age |  | -1.62 (0.51)  (P=0.001) | -1.70 (0.56)  (P=0.002) |  | -2.23 (0.47)  (P<0.001) | -2.21 (0.51)  (P <0.001) |
| Height |  |  | 0.06 (0.31)  (P=0.85) |  |  | 0.02 (0.29)  (P=0.93) |
| Alcohol consumption |  |  | -0.01 (0.07)  (P=0.85) |  |  | -0.10 (0.07)  (P=0.15) |
| Emphysema index (%) |  |  | -0.68 (0.63)  (P=0.29) |  |  | -0.47 (0.58)  (P=0.42) |
| **Independent**  **Variables** | **≥65 years old** | | | | | |
|  | **Unadjusted** | **Model 1** | **Model 2** | **Unadjusted** | **Model 1** | **Model 2** |
|  | **β (SE)** | **β (SE)** | **β (SE)** | **β (SE)** | **β (SE)** | **β (SE)** |
| Smoking  status | -6.49 (5.62)  (P=0.25) | -8.07 (5.55)  (P=0.15) | -8.68 (6.08)  (P=0.15) | -11.94 (5.28)  (P=0.02) | -13.44 (5.21)  (P=0.01) | -13.82 (5.75)  (P=0.02) |
| Age |  | -1.85 (0.56)  (P=0.001) | -2.05 (0.60)  (P <0.001) |  | -1.84 (0.52)  (P<0.001) | -1.92 (0.57)  (P <0.001) |
| Height |  |  | -0.65 (0.48)  (P=0.18) |  |  | -0.74 (0.46)  (P=0.11) |
| Alcohol consumption |  |  | -0.05 (0.15)  (P=0.73) |  |  | 0.04 (0.14)  (P=0.80) |
| Emphysema index (%) |  |  | -0.90 (0.90)  (P=0.32) |  |  | -0.59 (0.86)  (P=0.49) |

Note-CTBD, CT bone density; SE, standard error

Model 1 adjusted for age; Model 2 adjusted for age, height, alcohol consumption, and emphysema index.
